# Improving Fidelity of Translation of the Stepping On Falls Prevention Program through Root Cause Analysis

**DOI:** 10.3389/fpubh.2016.00251

**Published:** 2016-11-14

**Authors:** Jane E. Mahoney, Vicki L. Gobel, Terry Shea, Jodi Janczewski, Sandy Cech, Lindy Clemson

**Affiliations:** ^1^Department of Medicine, Division of Geriatrics and Gerontology, University of Wisconsin School of Medicine and Public Health, Madison, WI, USA; ^2^UW Health Department of Orthopedics and Rehabilitation, Madison, WI, USA; ^3^The Greater Wisconsin Agency on Aging Resources, Inc., Madison, WI, USA; ^4^Ageing, Work and Health Research Unit, Faculty of Health Sciences, University of Sydney, Sydney, NSW, Australia

**Keywords:** root cause analysis, falls prevention, dissemination, implementation, Stepping On

## Abstract

**Background:**

Fidelity monitoring is essential with implementation of complex health interventions, but there is little description of how to use results of fidelity monitoring to improve the draft program package prior to widespread dissemination. Root cause analysis (RCA) provides a systematic approach to identifying underlying causes and devising solutions to prevent errors in complex processes. Its use has not been described in implementation science.

**Methods:**

Stepping On (SO) is a small group, community-based intervention that has been shown to reduce falls by 31%. To prepare SO for widespread U.S. dissemination, we conducted a pilot of the draft program package, monitoring the seven SO sessions for fidelity of program delivery and assessing participant receipt and enactment through participant interviews after the workshop. Lapses to fidelity in program delivery, receipt, and enactment were identified. We performed a RCA to identify underlying causes of, and solutions to, such lapses, with the goal of preventing fidelity lapses with widespread dissemination.

**Results:**

Lapses to fidelity in program delivery were in the domains of group leader’s role, use of adult learning principles, and introducing and upgrading the exercises. Lapses in fidelity of participant receipt and enactment included lack of knowledge about balance exercises and reduced adherence to frequency of exercise practice and advancement of exercise. Root causes related to leader training and background, site characteristics and capacity, and participant frailty and expectations prior to starting the program. The RCA resulted in changes to the program manual, the training program, and training manual for new leaders, and to the methods for and criteria for participant and leader recruitment. A Site Implementation Guide was created to provide information to sites interested in the program.

**Conclusion:**

Disseminating complex interventions can be done more smoothly by first using a systematic quality improvement technique, such as the RCA, to identify how lapses in fidelity occur during the earliest stages of implementation. This technique can also help bring about solutions to these lapses of fidelity prior to widespread dissemination across multiple domain lapses.

## Introduction

The Centers for Disease Control and Prevention (CDC) developed the “Replicating Effective Programs” (REP) framework in 1996 to guide the process by which proven interventions may be translated into practice (1). Originally developed to guide dissemination of HIV prevention interventions (2–4), the REP framework has been used with a number of other interventions (1, 5). The REP framework conceives dissemination as occurring through four stages: precondition (where a draft package is developed), pre-implementation (where a draft package is pilot tested), implementation (where there is wider dissemination with simultaneous further feedback and refinement), and maintenance (where dissemination continues with further refinement as needed). However, the framework provides little information on how to refine the package at each stage while maintaining faithfulness to the original design.

Fidelity in implementation science is defined as the “the degree to which … programs are implemented … as intended by the program developers” (6). Fidelity can be measured in terms of delivery of, and participants’ receipt and enactment of, the key elements of a program (7–9). Monitoring fidelity is essential in the early phase of dissemination, when an intervention is being refined for widespread use (1, 9–12). During a randomized trial, training of intervention providers is likely to be intense and result in high quality fidelity. However, with packaging for widespread use, provider training may be less intense, and fidelity monitoring “in the field” may be of lower quality or non-existent. Therefore, as a package is developed for dissemination, it becomes critical to understand how an intervention may lose fidelity, referred to as “voltage drop.” The higher the complexity, the more likely it is that an intervention will suffer from “voltage drop” (13, 14). One way to prevent “voltage drop” with dissemination is to implement a draft program in a non-research setting, identify lapses to fidelity, then refine the program package with the intent of preventing such lapses in the future.

While there is substantial literature describing the importance of fidelity monitoring for implementation, there is little description of how to actually use results of fidelity monitoring to improve the draft program package (1, 15–17). For example, in the REP framework, Kilbourne et al. recommend that the draft package be pilot tested to assess feasibility, acceptance, and any implementation barriers, so that it can be refined based on that input (1). But no guidance is given on how to determine such refinements. Another frequently used framework, the Consolidated Framework for Implementation Research, states that executing, evaluating, and reflecting on a series of pilot implementations is integral to translating an intervention into practice. Reflection may include group and personal reflection but recommends no methodology to systematically guide reflection (18).

Six sigma is an engineering management strategy designed to improve quality and efficiency of operational processes. Designed by Motorola in 1986, it has been widely used across a variety of industries, including health care, to improve processes (19–21). Its primary components are define, measure, analyze, improve, control (DMAIC). The “analyze” component frequently utilizes root cause analysis (RCA). RCA provides a systematic approach to identifying underlying causes of errors in complex processes and devising solutions to prevent such errors in the future. It may play an important role in dissemination and implementation science, providing a methodology to systematically identify causes of, and solutions to, fidelity lapses with early implementation of a draft program package of an intervention. Its use could improve the reliability, consistency, and fidelity of widespread implementation of complex health behavior change interventions. The use of this approach in packaging a program for dissemination has not been described previously.

In 2007, the CDC funded a dissemination research study to prepare the Stepping On (SO) falls prevention intervention for widespread implementation. Developed in Australia, SO is a small group, community-based program that in a randomized trial decreased falls among high risk older adults by 31% (22). The program is based on adult learning and behavior change principles that build self-efficacy. It is facilitated by a leader who has training and experience in health care or gerontology. In seven weekly sessions, a home visit and a booster session 3 months after the program has concluded, the intervention uses a multiple risk factor approach to falls reduction through education, brainstorming, and problem solving. Workshop participants learn about risk factors from invited experts, practice balance and strength exercises that advance in difficulty, and discuss strategies to prevent falls. It is a complex intervention with many opportunities for “voltage drop” in fidelity. This qualitative research study describes, to our knowledge, the first application of RCA to improve dissemination and implementation of behavior change interventions. We describe how, with pilot implementation of the program, we identified lapses to fidelity in program delivery, and in participant receipt and enactment, assessed causes through a systematic process (RCA), and improved the program package for training and disseminating SO, with the goal of creating a high-fidelity package for national dissemination.

## Materials and Methods

Prior to this study, we had determined key elements of SO using a modified Delphi Consensus. The Delphi panel identified 85 key elements across the nine domains of adult learning, program components, role of group leader, role of peer coleader, exercise (starting and advancing), training and background of group leader, qualifications of invited exercise experts, home visit, and booster session.

After elucidating key elements, content experts (Jane E. Mahoney, Terry Shea, and Sandy Cech) in collaboration with the program developer (Lindy Clemson) prepared a draft program package for U.S. implementation. This package consisted of a training manual, used by the master trainer to train new leaders, and a program manual, used by the leader to implement the program (23). Both manuals were modified from the Australian originals to suit U.S. audiences. The lead Wisconsin trainer (Sandy Cech), who had 3 years of prior experience in implementing SO in the U.S., trained a registered nurse (RN) over 4 days to implement the program. The RN was employed by the senior apartment complex hosting the program.

The workshop was held in one of five senior apartment buildings owned by Lincoln Lutheran of Racine, Inc., a faith-based non-profit organization. Inclusion criteria for the workshop were age 65 and over, living in one of two adjacent apartment buildings in the apartment complex, and a history of one or more falls in the past year or a fear of falling. Exclusion criteria were cognitive impairment as judged by the Services Manager and planned absence from more than one of the sessions. Eligible seniors were invited to participate in the workshop by the Apartment Services Manager. Thirteen older adults were invited to participate, and two of these declined. Eleven seniors gave informed consent and were enrolled in the workshop. Human subjects’ approval was obtained from the University of Wisconsin Health Sciences Institutional Review Board.

### Fidelity

Fidelity of implementation was assessed for three areas: program delivery, participant receipt, and participant engagement (8, 9).

### Fidelity of Delivery

Content experts (Terry Shea, Jane E. Mahoney, and Sandy Cech) developed a tool to be utilized by an expert observer to measure fidelity of delivery of the intervention in each of the seven sessions, based on the key elements identified through the modified Delphi Consensus. The fidelity tool assessed whether specific program activities occurred using a yes/no scale. It also assessed the quality with which key elements were incorporated using a scale of excellent, very good, average, not adequate. For example, for the item, “The leader linked exercises to function,” it was rated for occurrence (yes/no) and if it occurred, for quality (excellent, very good, satisfactory, not satisfactory). Some key elements were judged in the context of specific activities (e.g., brainstorming about benefits of exercise, starting and upgrading balance exercises); others were rated for the session as a whole (e.g., leader facilitates engagement of all members of group). One item rated the degree to which the leader was teacher-like (poor fidelity) versus facilitator-like (high fidelity) using a 10-point scale. At the end of the tool, the expert observer was asked “What, if any, sections did you feel didn’t have the time managed well? If so, why? Was anything omitted, and what? Please note here anything of concern.”

To reduce burden on the expert observer, each key element was assessed for fidelity in at least one session. While some elements were assessed at multiple sessions, none were assessed at all sessions. Two expert observers, a peer coleader, and a physical therapist (PT) evaluated fidelity. The peer coleader was a retired RN who was a participant in SO 3 years prior, and who then served as peer coleader for at least one SO workshop per year for 3 years, and as a co-trainer for at least one leader training per year for 2 years. She observed fidelity of non-exercise events. A PT with professional experience working with seniors observed fidelity of exercise events.

### Fidelity of Participant Receipt and Enactment Related to Exercise

Stepping On is a multifaceted falls prevention program, with participants working on alleviating the falls risk factors that apply to them. For some, this may relate to low vision and the need to see an ophthalmologist; for others, modifications of medications may be important. However, all participants can benefit from improving balance and strength and so are expected to practice balance and strength exercises on a regular basis at home and advance them in difficulty. Because exercise enactment is important for all, we selected this element as the focus for the evaluation of fidelity of participant receipt and enactment.

In SO, a guest PT attends sessions 1, 2, and 6 to teach participants seven balance and strength exercises. Participants practice the exercises as a group in each of the seven workshop sessions, advancing as they are able, with guidance from the PT and workshop leader. In addition, participants are provided with an exercise manual and instructed to practice the exercises at home, daily for balance exercises and three times per week for strength exercises, advancing the level of difficulty at home as able. They are expected to continue exercising after the workshop ends.

To evaluate fidelity of participant receipt and enactment related to exercise, two trained researchers interviewed participants in the home during the week after the final session to ascertain exercise knowledge (receipt), and their adherence to home exercise practice, degree of advancement of exercise by self-report, and belief in exercise to prevent falls (enactment). The interviewer showed each participant a picture of each exercise and asked how it was helpful for them, if they were performing that exercise, and if so, how often in a week, and if not, why not. They were asked to demonstrate how they perform the exercise, and rate on a scale of 1–10, how much they thought exercise could play a role in preventing their falls.

### Other Data

Participants were assessed before the workshop for baseline demographics, self-report of use of assistive devices, number of falls in the year prior, and physical performance on the Timed Up and Go (24). Also before the workshop, survey data were obtained from the SO leader, the site coordinator, and invited experts (PT, pharmacist, low vision expert, police officer) to elicit their understanding of SO concepts, their belief in the benefit of SO to participants, and their self-efficacy to fulfill their role in SO. During the workshop, the SO leader completed a field log for each session about what worked and what did not work. After the SO workshop, the leader, site coordinator, and invited experts were surveyed again to evaluate their belief in the benefit of SO to participants, their self-efficacy to fulfill their role in SO, their preparation for their role in SO, and barriers they encountered in fulfilling their role in SO. In addition, a research assistant interviewed the leader, peer leader, site coordinator, and guest experts by phone using open-ended and semi-structured questions. The purpose of the phone interview was to explore in more depth the stakeholders’ perceptions of the program, their role in it, and any barriers to performance of their roles. Stakeholders were asked what they liked and did not like about the program and their role in it, what worked and what did not, and what they had expected their role would entail. Additional questions followed up on the stakeholders’ survey answers to understand, if a program component was not used or was difficult to use, why that was so, and what modifications were made.

### Analysis: Program Delivery

To identify lapses of fidelity in program delivery, data on fidelity observations of workshop sessions were reviewed by Jane E. Mahoney and Vicki Gobel. Expert observers’ notes were reviewed to gain insights on why the expert observer assigned a score of “did not occur” or “not satisfactory.” Jane E. Mahoney and Vicki Gobel each compiled lists of fidelity lapses separately then met to ensure all lapses were identified. Differences were adjudicated by jointly reviewing pertinent fidelity observations of workshop sessions. Lapse of fidelity in program delivery of a key element was defined as a score by the expert observer of “did not occur” or “not satisfactory” on the workshop fidelity tool. A leader being rated as more teacher-like than facilitator-like was also considered a lapse of fidelity. Lapses in fidelity in program delivery were categorized according to the domain of key elements to which they applied: program aspects, exercise, upgrading exercise, group leader’s role, background of group leader and peer coleader, for a total of seven domains regarding fidelity of delivery.

### Analysis: Participant Receipt and Enactment

Data from participant interviews post-session seven were used to investigate lapses in fidelity of participant receipt and enactment related to the key element domain of exercise. Each reviewer (Jane E. Mahoney and Vicki Gobel) coded the data separately to identify lapses and then met to adjudicate differences by referring back to the raw data. Lapse in participant receipt was defined as being present if 30% of participants lacked knowledge regarding correct frequency of exercises at the post-session seven interview. Lapses in participant enactment were defined as 30% of participants practicing exercises less frequently than prescribed, not practicing all the exercises, not advancing with balance and strength exercises by self-report, or not believing exercises will help. The thresholds of 30% were established by the research team’s context experts (Jane E. Mahoney, Sandy Cech, Terry Shea, and Lindy Clemson), based on Lindy Clemson’s findings from the original SO study.

### Analysis: Other Data

Following coding of fidelity data, Jane E. Mahoney and Vicki Gobel reviewed the field logs of SO leaders, notes from expert observers, and all interviews and surveys of participants, SO leaders, site coordinators, and guest experts to become familiar with the materials. These data were not coded prior to the RCA; rather they were used as raw material and referred back to during the RCA as a form of reflective validation.

### Root Cause Analysis

We used the RCA process to identify underlying causes of lapses to fidelity in delivery, receipt, or enactment. RCA is a method that is often used as part of DMAIC to address a problem from a systems approach, using the “5 whys” technique” (25–28). It involves working backwards from the problem by continuing to ask why it happened, until you find one or more “root causes.” These are then defined as the causes, and if corrected, they should keep the problem from recurring. The RCA team typically includes content experts and stakeholders from the site where the problem occurred. The RCA process may utilize a fishbone diagram (29), where the bones of the fish are considered as the categories of inquiry, with causes elicited from “asking why five times” becoming subcategories under each bone. The first procedure when using a fishbone diagram is for the RCA team to determine the categories of possible causes (i.e., the bones of the fishbone). While standard categories are available for health care and industry (e.g., policies; procedures; people; plant/technology), each team is expected to determine the categories needed for their subject matter (30). Once the categories of inquiry are defined, the team proceeds to brainstorm possible causes and attach them to the appropriate branch, continuing to ask why for each possible cause, until all root causes are identified.

We convened an RCA team of three content experts: an MD (Jane E. Mahoney), a PT (Terry Shea), and an RN (Sandy Cech), three injury prevention research experts (two of whom had conducted participant interviews), and the research coordinator (Vicki Gobel). The group met in three sessions for a total of 10 h. Prior to the RCA sessions, team members received educational materials regarding the RCA process and a summary of all identified lapses of fidelity in delivery, receipt, and engagement. The RCA process began with group consensus to determine the categories of primary causes (i.e., the bones of the fishbone), defined as categories that would be further analyzed to ascertain potential root causes for all fidelity lapses. Next, the group brainstormed secondary and underlying causes for lapses of fidelity. To assist with identifying root causes, Vicki Gobel and Jane E. Mahoney provided findings from the surveys, interviews, and field logs of participants, leaders, site coordinators, and guest experts. For each root cause proposed by the RCA team, Jane E. Mahoney and Vicki Gobel reviewed the primary data to verify mention of that cause. For example, a potential root cause could be “participant was too frail to benefit from group exercise,” which had been elicited from participant pre-surveys and leader and PT interviews. If the primary data did not support that as a proposed root cause, then it was deleted.

The RCA process was conducted for all lapses in delivery, covering one domain at a time, until all seven domains were investigated. For example, lapses in the domain of exercise delivery could have primary causes in five different categories: participants, site and support, leader background, leader training, and the exercises themselves. For each category of primary cause, the group used the “ask why five times” technique to identify underlying root causes for lapses in fidelity of delivery in that domain. The RCA process was likewise conducted for lapses in participant receipt and engagement in the domain of exercise. Table 1 describes the steps of the RCA process, and the inputs, and outputs at each step.

**Table 1:** **Steps of root cause analysis**.

RCA step	Inputs	Outputs
Determine lapses of fidelity and categorize by domains	Delphi consensus to determine key element domains	Table 3: list of lapses in fidelity of delivery for 7 key element domains fidelity of receipt for key element domain of exercise fidelity of enactment for key element domain of exercise
	Fidelity observations of sessions to determine lapses in fidelity of delivery	
	Participant interview post-session 7 (fidelity of exercise receipt, fidelity of exercise enactment)	
Populate fishbone diagram with categories of primary causes to be used for RCAs	RCA team consensus regarding the categories of possible causes for fidelity lapses (i.e., bones of fish)	Bones of fish to be used with RCAs for lapses of fidelity in delivery, receipt, and enactment, by key element domain
For each RCA, brainstorm possible causes using 5-why’s technique	RCA team	Preliminary fishbone diagram for lapses of fidelity in delivery, receipt, and enactment, by key element domain
Verify root causes	RCA team members’ review of primary data: field logs of Stepping On leaders notes from expert observers interviews and surveys of participants, Stepping On leaders, site coordinators, and guest experts	Completed fishbone diagrams with root causes of lapses in fidelity of delivery, receipt, and enactment, by key element domain
		Table 4: summary of root causes by key element domain
Identify solutions	Program developer and content experts on Stepping On research team	Table 5: changes made to Stepping On program based on RCA

Following elucidation of root causes, the PI (Jane E. Mahoney) met with the content experts and the program’s developer (Lindy Clemson) over the course of 1 month to develop solutions for each root cause.

## Results

Characteristics of the 11 participants enrolled in the SO workshop are shown in Table 2. Most were females, fewer than half had been educated beyond high school, and most had fallen in the last year. There were two husband–wife couples in the group. The mean of the timed up and go physical performance measure indicated high risk for falls (31).

**Table 2:** **Characteristics of subjects in pilot Stepping On workshop (*n* = 11)**.

Characteristic	Mean (SD) or *n* (%)
Age, *m* (SD)	86 (4.4)
Gender, female, *n* (%)	8/11 (72%)
Education, *n* (%)	
-beyond high school	4 (36%)
-high school	5 (45%)
-less than high school	2 (18%)
Race/ethnic group, *n* (%)	
-Caucasian	11 (100%)
-African American	0/11 (0%)
-Latino	0/11 (0%)
Use of assistive device for walking, *n* (%)	5 (45%)
Fallen in the past year, *n* (%)	9 (82%)
# falls in the past year, *m* (SD)	1.4 (1.6)
Timed up and go, *m* (SD)^a^	19.84 (8.33)

*^a^Timed up and go of >13.5 indicates high risk for falls (32)*.

Table 3 shows lapses in fidelity of delivery by domains of key elements. Most of the lapses were in the domains of group leader’s role, use of adult learning principles, and in introducing and upgrading the exercises. In the domain of leader role, the leader lacked adequate skill guiding the guest expert, did not foster discussion or sharing of stories, and lacked adequate skill in reflective listening. Lapses in use of adult learning principles included limited or inadequate use of the following: brainstorming, the prevention framework to problem solve falls, and of facilitating participant question and answers and discussion. Exercise lapses included inadequate linkage of exercises to how they prevent falls, not using weights and not advancing exercises. In general, the leader tended to function more as a teacher than a facilitator.

**Table 3:** **Lapses in fidelity of delivery, receipt, and enactment of key elements of Stepping On according to key element domain**.

Key element domain	Lapse in fidelity identified by expert observation at one or more sessions or at post-session seven participant interview
**Delivery**	
Adult learning	1. Brainstorming insufficient or not done where indicated in manual 2. Time for questions not always provided; questions not always encouraged 3. Insufficient facilitation of discussion (e.g., how to accomplish exercise at home, how to identify safe shoes) 4. Insufficient or poor quality group problem solving on how to prevent falls or accomplish exercise (e.g., “prevention framework”) 5. Did not link content to participants’ personal stories 6. Participants shared few stories on advancing exercises and remembering to do exercises 7. Participants not asked what they want to cover in final session
Program	8. Some key activities omitted 9. Handouts given out all at once rather than with each activity
Exercise	10. Exercises not performed safely 11. Leader, guest therapist did not stress importance of doing exercises in standing position 12. Did not practice all exercises in session two 13. Exercises not linked to how they prevent falls 14. Leader did not review frequency of balance and strength exercises 15. Did not collect exercise log
Upgrading exercise	16. Leader did not ask if anyone would like to demonstrate how to advance exercises 17. Leader did not offer and encourage weights with exercise practice 18. Leader did not discuss how to advance strength exercises 19. Leader and PT did not satisfactorily encourage participants to advance balance and strength exercises
Group leader role	20. Leader did not inquire about needs relate to vision or hearing impairment 21. Did not prompt guest expert to deliver correct content and break down content into simple steps 22. Did not demonstrate skill in storytelling 23. Did not facilitate/prompt stories from participants 24. Did not demonstrate skill in reflective listening
Leader training and background	25. Demonstrated poor knowledge of fall prevention topics necessary for session
	26. Functioned more as a teacher than as a facilitator
Peer coleader role	27. Peer coleader did not prompt participants to ask questions 28. Peer coleader poorly modeled how to be active participant
**Receipt**	
Exercise	29. Participants lacked knowledge of correct exercise frequency
**Enactment**	
Exercise	30. Participants did not practice all exercises 31. Participants practiced exercises at less than recommended frequency 32. Participants lacked belief in importance of exercise

Lapses in fidelity of participant receipt and enactment in the key element domain of exercise are also shown in Table 2. Also, 6 (55%) of 11 participants did not know the correct frequency of practice for strength exercises (receipt). For engagement, four (36%) did not adhere to practice of all exercise, seven (64%) did not adhere to prescribed frequency of practice, four (36%) lacked belief that exercises would help, and six (55%) did not advance in level of challenge with balance or strength exercises.

**Figure 1:**
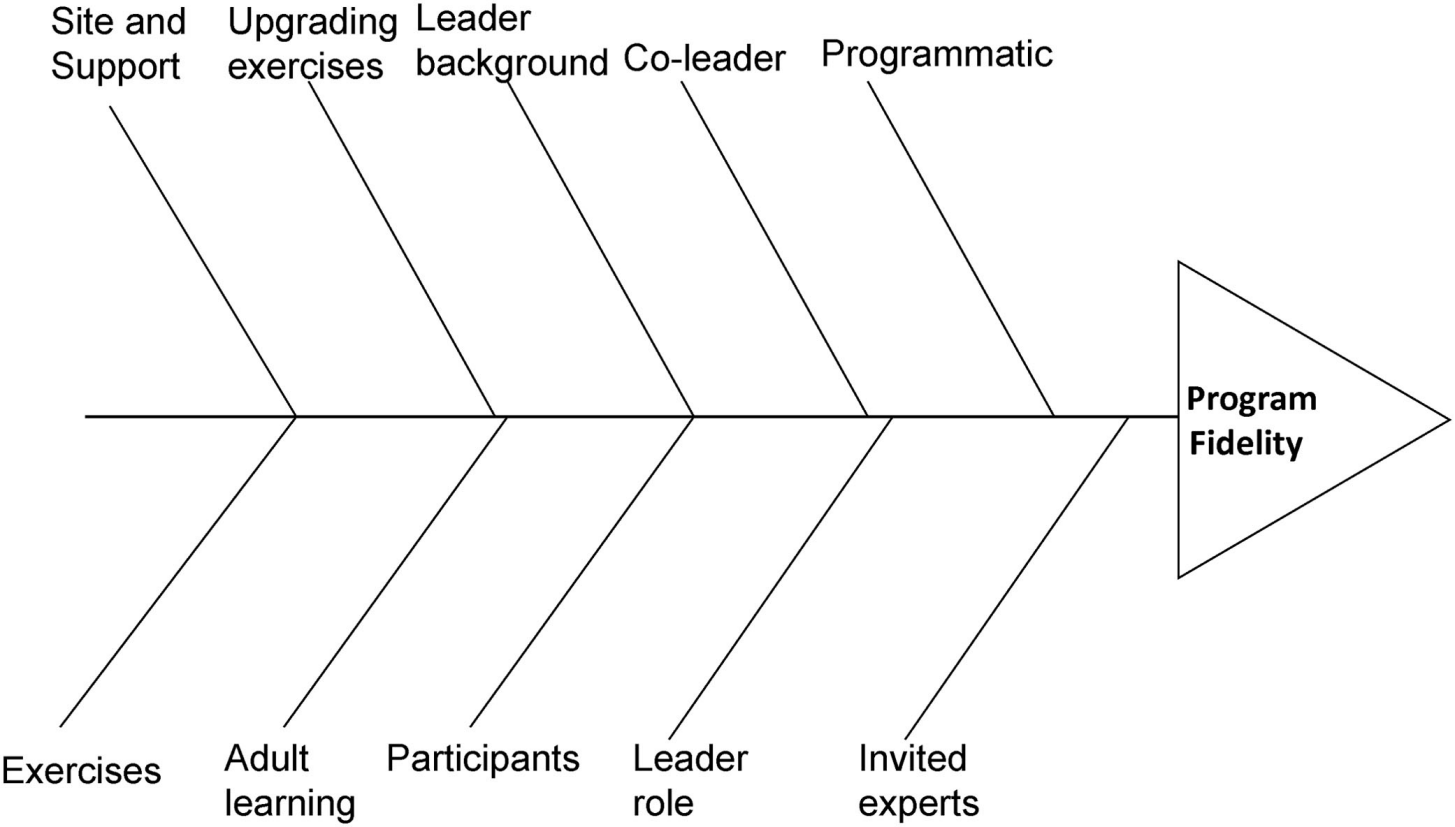
**Categories selected for fishbone framework for root cause analysis**.

Figure 1 shows the fishbone diagram with primary categories within which we looked for underlying causes of lapses of fidelity. Primary categories included those key to the program: adult learning, program content and activities, exercise and upgrading exercises, group leader role, leader background and characteristics, peer coleader role, and invited experts. Two additional categories, “participants” and “site and support,” were added as they could contribute underlying causes.

Table 4 summarizes root causes for each key element domain in which there were fidelity lapses. For each domain, there were multiple root causes that originated from multiple categories of the fishbone diagram. For example, fidelity lapses in the domain of exercise had causes related to leader role, site and support, participants, and invited experts. Within the fishbone category of “leader role,” root causes included insufficient leader training, practice, and feedback on how to teach older adults to perform and advance exercises, and on how to work with the invited PT. Within the category “site and support,” root causes included insufficient information provided to the site regarding how to recruit participants and who should be recruited, with the result that site coordinator recommended the program to the most mobility-impaired residents and potentially oversold the program’s benefits. Within the category of “participants,” participants may have been too frail to advance and may not have been motivated to exercise. Within the category “invited expert,” the PT may not have been sufficiently prepared ahead of time for his/her role. Root causes of lapses of key elements in other domains similarly mapped to multiple categories of the fishbone.

**Table 4:** **Root causes of lapses in fidelity of delivery, receipt, and engagement by key element domain**.

Key element domain of fidelity lapses	Root causes
Adult learning	Leader lacked experience in facilitation and behavior change Training did not sufficiently emphasize adult learning, did not provide enough opportunity for leader to practice with feedback Sessions had too much content; leader may not have understood to prioritize adult learning principles Manual and training did not sufficiently emphasize importance of establishing trust in session one Site appointed person to be leader; leader may have lacked motivation Leader had other roles at site; may have lacked time to prepare
Program	Too much content for education level and frailty of group Group size too large for frailty of group Training and manual did not emphasize which activities and elements were key Handouts were overemphasized in manual and training Too many handouts; manual lacked guidance on which were required vs. optional Leader and site coordinator had other demands on time and may not have communicated well regarding preparation of handouts Training and manual did not clearly explain about communication with site coordinator Site did not understand time required to run program Program may not have been good fit related to site’s mission
Exercise and upgrading exercise^a^	Training and manual did not emphasize leader mastery of practice and advancement of exercises; leader not required to demonstrate mastery Leader may have lacked belief in importance of advancing exercise Participants may have been too frail for group exercise and advancement Manual and training did not explain how sites should screen participants Program had no criteria for who would be too frail to participate Training and manual did not emphasize key elements related to exercise Site coordinator did not adequately explain program to participants; participants may have had too high expectations at outset Leader did not stress safety and slow advancement (at your own pace) Leader lacked sufficient training to have self-efficacy to prompt invited physical therapist to manage time and stress key elements Site coordinator did not sufficiently prepare invited physical therapist ahead of time
Leader role	Leader lacked prior experience in behavior change group facilitation Goals of storytelling were not clearly articulated; leader training and manual did not emphasize, and training did not provide practice in storytelling Manual did not indicate which elements/activities were key Manual lacked cues to prompt invited expert Training lacked sufficient emphasis on, and practice with feedback on how to work with guest expert, facilitate group, engage in reflective listening Too much program content may have prevented facilitation, reflective listening, storytelling
Leader background	Site managers not briefed sufficiently on importance of facilitation experience and motivation for potential leader Site manager not briefed sufficiently on amount of leader time needed to accomplish workshop
Peer coleader	Site and leader did not have sufficient knowledge before workshop on how to select peer coleader Training and manual did not emphasize how to train peer coleaders, importance of, and how to debrief with peer coleader after each session Training did not provide practice on how to give feedback to peer coleader

*^a^Key element domains for exercise and upgrading exercise were combined as they shared root causes*.

Table 5 summarizes changes made as a result of the RCA. Changes were made to the SO program manual, to the training program, and training manual for new leaders and to the methods for and criteria for participant and leader recruitment. A Site Implementation Guide was created to provide information ahead of time to sites interested in implementing SO.

**Table 5:** **Changes made to Stepping On program package as a result of root cause analysis**.

Program package area	Changes made
Program and program manual	Modified program Decreased number of handouts, changed some handouts to references on display table Simplified some content areas Clarified communication between leader and site coordinator regarding distribution of handouts (give out and go over after group discussion) Increased information about how to start and progress exercises Increased information in Participant Exercise Manual about when to advance Provided more specific cues to leader to prompt for questions, cue invited expert to manage time, facilitate brainstorming, etc. Added “key” symbol in manual next to important components
Leader training	Modified training Increased didactics, discussion, practice, and group and master trainer feedback on practice for the following areas: ○ group facilitation ○ starting and upgrading exercise ○ principles of adult learning ○ role of session one in developing trust Open-book quiz to assess falls knowledge Key elements quiz By end of training, must demonstrate skill at leading and upgrading exercise, and leading small group Stepping On activity Increased emphasis on communication with site coordinator More information on peer coleader role and how to recruit and train peer coleader Post-training feedback provided by master trainer based on fidelity check of any of sessions two to six of leader’s first workshop Leader self-evaluation tool for sessions three and six
Leader background	Changed leader application form and screening process Ensure leader has prior experience with adult small group facilitation
Information for prospective sites	Created Site Implementation Guide with information Qualifications of leader, peer coleader Roles of leader, site coordinator Activities, time, and cost Criteria for recruitment of older adults
Participant recruitment and enrollment	Established new criteria Willing to engage in group activities and home exercise Exclude older adults who require a walker for indoor walking Decrease group size to eight to ten if there are high proportion of participants who use assistive devices Prep physical therapist ahead of first workshop regarding frailty level of group Created participant screening and enrollment form

## Discussion

To our knowledge, this is the first application of RCA in dissemination and implementation research. Using RCA, we identified causes and developed solutions to lapses to fidelity that occurred with the first implementation of a program package for SO. Following the six sigma approach (DMAIC), we defined key elements, measured fidelity with those elements in intervention delivery, receipt, and enactment, analyzed root causes, and improved the draft package for widespread dissemination. The RCA allowed us to get beyond a simplistic primary cause (i.e., “leader insufficiently trained”) to understand the contribution of complex, interacting human and system factors. We identified that organizational knowledge and readiness, leader background and competing tasks, and participants’ levels of frailty all contributed to lapses in fidelity of delivery, receipt, and enactment.

A program package for dissemination of a complex intervention may include a number of components: a provider protocol, a training program and materials for providers, recruitment criteria and guidelines, forms and materials for participants, and an implementation guide and materials for the organization hosting the intervention. While all these components are often necessary for dissemination, not all may be developed as part of the original randomized trial (1). Our study shows that early monitoring for fidelity of implementation with a draft program package can help identify, create, and refine components needed for broad dissemination. Here, the RCA of SO implementation led to changes in the program manual, participant handouts, leader training, fidelity monitoring, participant enrollment criteria and process, and communication process between site coordinator and workshop leader. The types of changes varied. Some were very simple, such as attention to group size, and others were more complex, such as making sure that by the end of training leaders understood the broader concepts behind the program, like as how to engage older people in learning and behavior change. Others were at an administrative and organizational level, such as changing how prospective sites should be informed about the program. The diversity of changes to the program package (from information provided to sites, to the program manual, to who can lead the intervention and the type of training they need, and to who should participate in the program) can be attributed to the systems approach intrinsic to RCA. Such a systems approach is necessary to create a program package that will lead to consistent high-fidelity implementation by a wide variety of organizations.

Measuring fidelity of implementation is essential to maintaining quality and effectiveness of behavior change interventions (1, 9–12, 33, 34). While there is consensus on the importance of fidelity, there is scant research examining how to use findings of poor fidelity to improve a draft program package before widespread dissemination. Gearing et al. found that out of 24 peer-reviewed articles examining implementation fidelity, only 1 discussed use of corrective feedback in any detail (35), and in only 4 was it mentioned or discussed moderately (36–39). In six conceptual papers on fidelity (9–12, 33, 34), feedback is explicitly mentioned as a construct of fidelity in only one (33), and in none is it described how to systematically use fidelity assessment to improve implementation. Yet this is obviously important, as the program package for widespread dissemination must result in a highly reproducible product. The DMAIC methodology provides a systematic way to identify and apply corrective feedback to improve the draft program package prior to widespread dissemination. We identified that lapses in fidelity with first implementation of the draft program can result in substantial changes.

The REP framework is a widely used framework to guide packaging of proven intervention for widespread dissemination through the stages of precondition, pre-implementation, implementation, and maintenance (1). However, it provides little guidance on how to make modifications at each stage while still ensuring fidelity. Our study enhances the REP framework, demonstrating the value of the DMAIC approach to maximize fidelity as an intervention moves from pre-implementation to implementation.

The DMAIC approach may be especially important when trying to bring complex interventions to scale. DMAIC and RCA approaches have been used frequently in health-care delivery systems to understand errors with complex processes and identify solutions. The RCA focuses not on active errors (i.e., error made by individuals that directly or indirectly caused the event), but rather on latent and environmental causes (i.e., organization-related and environment-related causes that predispose to active errors) (28). In our analysis, RCA allowed us to similarly focus on latent and environmental causes, and away from active errors (i.e., made by the leader or invited expert). Latent causes included those that could be remedied through changing the program manual, leader training, training manual, or leader background and recruitment. Environmental causes related to participants, in particular participant recruitment criteria. Environmental causes also related to communication patterns, roles and competing agendas of the sponsoring organization, site coordinator, and program leader. While the SO program cannot impact the competing agendas facing the sponsoring organization, site coordinator, or program leader, the RCA led to a number of changes to better inform sites of what the program would involve, allowing them to decide if SO would be a good fit for them.

There are a number of limitations to this study. First, we used the DMAIC methodology on only one pilot of the program. Other SO workshops may reveal other problems. Second, in order to decrease burden on the rater, raters did not observe fidelity to every key element in every session. As a result, we could not tabulate the total number of sessions in which a specific fidelity lapse occurred. It is possible that an element was delivered with adequate fidelity at a session where it was rated, but not at another session (where it was not rated) or *vice versa*. Third, the fidelity tool was used by two expert observers, each of whom examined elements within their expertise. Further testing of the tool, including inter-rater reliability testing, is necessary before widespread use. Fourth, the leader was a novice and had little chance to practice new skills. It may be that experience would negate these lapses in fidelity. However, it is more likely that the outcomes of this process, in particular the enhanced training and coaching that resulted, would serve to accelerate novice leaders to expert. Fifth, there are inherent biases in any causal analysis of adverse events (32, 40). To decrease judgment bias and recognition bias, we used a multidisciplinary team comprising SO content experts, physical therapy and geriatric physician falls experts, and injury prevention research experts, spent sufficient time at the outset to brainstorm the field of potential causes (i.e., the causal field), and avoided time constraints on analysis. However, some bias remains due to the fact that the analysis occurred after all ancillary data were collected. During an RCA, analysis of causes may prompt additional data collection; we were not able to go back to study subjects (participants, leader, invited expert, site coordinator) to gather additional data during the RCA. Sixth, our study does not report on fidelity of delivery of SO with later REP framework stages of implementation and maintenance. Monitoring of fidelity in implementation and maintenance stages of program dissemination is similar to the *control* phase of DMAIC, with the goal being to ensure the package is implemented widely and over time with high quality.

In summary, when translating complex interventions, we suggest that it is essential to use a proven quality improvement technique such as DMAIC and RCA at the pre-implementation stage, to refine the program prior to widespread use. Importantly, as can be seen in this study, the RCA allows identification of multiple domains of causes, rather than focusing on a simplistic solution of “provide more training.”

## Author Notes

JM is board certified in geriatrics and internal medicine. She is a Professor of Geriatrics in the University of Wisconsin School of Medicine and Public Health. She also serves as Executive Director of Wisconsin Institute for Healthy Aging, a non-profit organization that disseminates evidence-based prevention programs for older adults. She is Principal Investigator of the Community-Academic Aging Research Network, an NIA-funded initiative to support research collaboration between University of Wisconsin researchers and community partners from Wisconsin’s Aging Network. Dr. JM has received funding from the American Physical Therapy Foundation, the CDC, the NIA, and the State of Wisconsin for epidemiologic and clinical research on falls. She has studied risk factors for falls after hospitalization, clinical trials of community-based multifactorial falls interventions, and dissemination research on the Stepping On falls prevention program. She is currently working with University of Wisconsin’s Active Aging Research Center to help develop internet-based technologies to help older adults reduce falls and maintain independence.

## Author Contributions

JM was responsible for conceptualizing the theoretical and empirical formulations of the research project, literature review, study protocol, and design, and collecting, analyzing, and interpreting data as well as manuscript preparation. VG was part of the original study group that participated in development of solutions based on findings of the RCA and provided feedback on draft versions of the paper. JJ participated in conduct of the root cause analysis and feedback on draft versions of the manuscript. TS and SC were also part of the original study group that prepared a draft program package for U.S. implementation. LC developed the program in Australia initially and helped the original study group prepare the program for U.S. implementation.

## Disclaimer

The content is solely the responsibility of the authors and does not necessarily represent the official views of the Centers for Disease Control and Prevention. Presented in part at the 65th Annual Meeting of the Gerontological Association of America, San Diego, CA, USA, November 2012.

## Conflict of Interest Statement

JM and LC are co-authors on the Stepping On Leader Manual, Third North American Edition, Frieburg Press, Cedar Falls, IA, USA, 2011. All the other authors declare no conflict of interest.

## Abbreviations

CDC, Centers for Disease Control and Prevention; DMAIC, define, measure, analyze, improve, control; MD, medical doctor; PT, physical therapist; RCA, route cause analysis; REP framework, replicating effective programs framework; RN, registered nurse; SO, stepping on.
